# Quality of life evaluation in Japanese pregnant women with striae gravidarum: A cross-sectional study

**DOI:** 10.1186/1756-0500-5-450

**Published:** 2012-08-21

**Authors:** Kotomi Yamaguchi, Nobuhiko Suganuma, Kazutomo Ohashi

**Affiliations:** 1 Department of Children and Women’s Health, Division of Health Sciences, Osaka University Graduate School of Medicine, 1-7 Yamadaoka, Suita, Osaka, 565-0871, Japan; 2 Department of Human Health Sciences, Kyoto University Graduate School of Medicine, 53 Kawahara-chou, Shogoin, Sakyou-ku, Kyoto, 606-8507, Japan

**Keywords:** Striae gravidarum, Quality of life, Japanese pregnant women

## Abstract

**Background:**

Striae gravidarum is a physiological skin change that many pregnant women experience during pregnancy. The striae are often accompanied by a reddish purple color during pregnancy, and then lose pigmentation and become atrophic in the long term after pregnancy. Striae gravidarum seems to be undesirable to many pregnant women. However, the impact of striae gravidarum on pregnant women who experience it has not been clarified. The aim of this study was to evaluate the impact of striae gravidarum on the generic and dermatology-specific quality of life (QOL) of pregnant women.

**Methods:**

A cross-sectional study was conducted at three private clinics in a typical urban area in Japan. We recruited 447 pregnant women at 36 weeks of gestation; One hundred and ninety-nine pregnant women at 36 weeks of gestation participated in the study and 179, consisting of 94 primiparae and 85 multiparae, were analyzed.

We used and assessed Davey’s score for striae gravidarum, World Health Organization Quality of Life assessment questionnaire for generic QOL, and Skindex-29 for dermatology-specific QOL.

**Results:**

The prevalence of striae gravidarum was 39.1% (27.7% in primiparae, and 51.8% in multiparae). Although there were no differences in generic QOL scores between the presence and absence of striae gravidarum and with their severity, the whole group of pregnant women and the multiparae group showed significant differences in scores on emotion of Skindex-29 between the presence and absence of striae gravidarum (*p* = 0.012 and *p* = 0.011). Pregnant women with severe striae gravidarum showed significantly higher scores on emotion of Skindex-29 compared with those with absent or mild striae gravidarum (*p* < 0.001 and *p* = 0.005).

**Conclusions:**

There was no difference in generic QOL of pregnant women between the presence and absence of striae gravidarum, although the occurrence and severity of striae gravidarum influenced their dermatology-specific QOL. Multiparae women were especially impaired by striae gravidarum and it is considered important to prevent or reduce the severity of striae gravidarum of the multiparae group.

## Background

Striae gravidarum is a physiological skin change that many pregnant women experience during pregnancy [1,2]. They are linear lesions frequently found on the abdomen, breasts, buttocks and thighs. They are often accompanied by a reddish purple color during pregnancy, and then lose pigmentation and become atrophic in the long term after pregnancy. As striae gravidarum are regarded as a physiological change, medical professionals do not pay much attention to pregnant women with this condition, and thus few data on the prevalence and QOL of women having striae gravidarum are available. However, striae gravidarum seems to be undesirable to many pregnant women.

Striae gravidarum do not pose a health risk. However, they are often symptomatic, causing itching and discomfort as well as psychological distress in pregnant women when severe. Additionally, striae gravidarum, especially on the abdomen, are a cause of cosmetic concern for pregnant or postpartum women, who often seek various expensive and painful treatments even if such treatments are not effective [1,2]. It thus seems to be important to objectively evaluate the influence of striae gravidarum on the QOL of pregnant woman and postpartum women.

Since a QOL evaluation is regarded as important in those dermatological conditions that do not threaten life, generic and dermatology-specific QOL evaluations are combined for research in dermatology. According to the review described by Both [3], the SF-36 and WHOQOL-BREF as generic scales, and the Skindex-29 and Skindex-17 as dermatology-specific scales are recommended for evaluating the QOL of patients with dermatological conditions. In the present study, Davey’s scoring [4] was used to evaluate the abdominal striae gravidarum of Japanese pregnant women. Also, the QOL of pregnant women was examined using WHOQOL-BREF and Skindex-29. We then determined the association between the severity of striae gravidarum and QOL. The aim of this study was to evaluate the impact of striae gravidarum on the generic and dermatology-specific quality of life (QOL) of pregnant women.

## Methods

### Design

A cross-sectional study design was adopted using a self-administered questionnaire. The study was performed at three private clinics in Aichi Prefecture between October 2010 and January 2011. Aichi Prefecture is a typical urban area of central Japan. The Ethical Committee of Osaka University Medical School approved the study. The anonymity of the participants was preserved.

### Participants and procedure

We recruited all pregnant women who visited for an antenatal examination at 36 weeks of gestation during the study period, excluding twin and threatened premature cases. Pregnant women were given an explanation of the content of the study by a document and orally during waiting for prenatal examination by researchers. Written informed consent regarding study cooperation was obtained from all participants.

Of the 447 pregnant women recruited, 199 (44.5%) agreed to participate in the study and provided written informed consent. The women were surveyed using the Skindex-29 and WHOQOL-BREF questionnaires. Data regarding age at delivery, maternal height, maternal weight at delivery and maternal weight before pregnancy were collected from their medical charts. After collecting the questionnaires, the striae gravidarum on the abdomen were assessed by three researchers (one in each clinic) according to Davey’s method.

Of the 199 participants, 20 were excluded from the study due to incomplete questionnaires in 17, no medical records in 2, and no record of Davey’s score in 1. The clinical characteristics of the 179 pregnant women, consisting of 94 primiparae and 85 multiparae, participating in this study, are summarized in Table 1. Recently, striae gravidarum was significantly more common in women who had been born prematurely than in women who had been born at term [5]. Then, pregnant women delivered after 37 weeks of gestation were used as subjects of the study.

**Table 1:** Clinical characteristics

	**Total**	**Primiparae**	**Multiparae**	***p-*value**
Age	30.8 ± 4.2 (n = 179)	29.9 ± 4.0 (n = 94)	31.8 ± 4.2 (n = 85)	0.002
	(21–42)	(23–40)	(21–42)	
Height	157.9 ± 5.7 (n = 179)	158.4 ± 5.9 (n = 94)	157.3 ± 5.2 (n = 85)	0.188
	(145.0-178.0)	(146.0-178.0)	(145.0-170.0)	
Weight before pregnancy	52.4 ± 8.3 (n = 178)	51.3 ± 7.4 (n = 94)	53.6 ± 9.1 (n = 84)	0.054
	(38.0-90.0)	(40.0-80.0)	(38.0-90.0)	
Weight at delivery	62.2 ± 8.5 (n = 177)	61.5 ± 7.8 (n = 93)	63.1 ± 9.1 (n = 84)	0.208
	(42.7-96.8)	(47.2-92.8)	(42.7-96.8)	
Weight gain during pregnancy	9.9 ± 3.1 (n = 176)	10.2 ± 3.1 (n = 93)	9.5 ± 3.2 (n = 83)	0.107
	(1.0-22.8)	(1.0-22.8)	(1.7-16.1)	
BMI before pregnancy	21.0 ± 3.2 (N = 178)	20.4 ± 2.6 (n = 94)	21.7 ± 3.6 (n = 84)	0.008
	(16.0-38.0)	(16.0-30.4)	(16.5-38.0)	
BMI at delivery	25.0 ± 3.1 (N = 177)	24.5 ± 2.6 (n = 93)	25.5 ± 3.5 (n = 84)	0.031
	(17.3-40.0)	(18.4-32.3)	(17.3-40.0)	

( − ) indicates in range.

Date were analyzed by unpaired *t*-test.

### WHOQOL-BREF

The WHOQOL-BREF was developed collaboratively in several countries including Japan [6,7]. It assesses a generic QOL that is applicable to people living in different cultures. The Japanese version has been developed with good reliability and validity [7,8].

The WHOQOL-BREF is a 26 item self-administered questionnaire and consists of 24 items in four domains: physical health, psychological, social relationships, environment, and two ‘benchmark’ items about general well-being. The participants selected responses ranging from 1 (none) to 5 (extremely). Scores were calculated according to the guidelines for the WHOQOL-BREF. Higher scores indicate higher QOL.

### Skindex-29

This questionnaire was developed by Chren et al. in 1997 and consists of 30 items forming three scales: emotion in 10 items, symptoms in 7 items, and functioning in 12 items (item 18 is not included in scoring the instrument) [9]. The emotions scale measures the psychological effects of the disease, such as anxiety of being seriously ill, anxiety of getting a scar, anxiety of getting worse, depression, ashamedness, embarrassment, anger, frustration, humiliation, or annoyance. The symptoms scale measures the physical burden of the disease, such as pain, burning/stinging, itching, fluid accumulation-induced anxiety, irritation, sensitiveness, or bleeding. The functioning subscale focuses on the changes in daily life, such as sleep, work/hobby, social life, how close one can be with loved ones, desire to be with people, affection, staying at home, a problem for the people one loves, interference with sex life, doing things by oneself, interaction with others, or tiredness. The participants can select responses ranging from 0 (never) to 4 (all the time). All responses were transformed into linear scale scores, which varied from 0 (no effect) to 100 (maximum effect). The three subscales score separately and a higher score indicates a lower QOL. The scale has been translated into German [10], Italian [11], Spanish [12] and Turkish [13]. The Japanese version of Skindex-29 was made by Fukuhara [14] and sufficient reliability was demonstrated with Cronbach’s alpha coefficiencies of 0.94 in emotion, 0.81 in symptoms, and 0.94 in functioning. The reproducibilities were r = 0.93, 0.91, and 0.91 in each subscale.

### Davey’s score

The abdomen was divided into quadrants using the midline and horizontal line through the umbilicus. Each quadrant was scored 0 (=clear skin), 1 (=moderate number of striae) or 2 (=many striae). Total sum scores ranged from 0 to 8. The severity of striae gravidarum was divided into three categories, 0 (absent), 1 to 2 (mild), and 3 to 8 (severe), as assessed by Davey’s score.

For the standardization of scoring for objectivity, a researcher (Yamaguchi) visited every clinic and educated the researchers to learn Davey’s score using the drawings in the manuscript written by Buchanan, et al. [15]. During the research, the rate of appearance of striae gravidarum was periodically compared among clinics.

### Statistical analysis

The data analysis was performed with IBM SPSS Statistics 19. The unpaired *t*-test was applied for statistical analyses of subject profiles. The chi-squared test was used to examine the relationship between Davey’s scores and parity. The QOL scores on the Skindex-29 and WHOQOL-BREF were analyzed using the Mann–Whitney test and the Kruskal-Wallis test followed by the Mann–Whitney test with Bonferroni correction. The significance level was set at 5%.

## Results

### Sample characteristics

The clinical characteristics of the 179 pregnant women, consisting of 94 primiparae and 85 multiparae participating in this study, are summarized in Table 1.

The severity of striae gravidarum was divided into three categories, 0 (absent), 1 to 2 (mild), and 3 to 8 (severe), as assessed by Davey’s score. The prevalences of striae gravidarum were 39.1% (70/179), 27.7% (26/94) and 51.8% (44/85) in the whole group of pregnant women, primiparae and multiparae, respectively (Table 2). The severity of striae gravidarum depended on parity (p < 0.001), and severe cases appeared in 4.3% of primiparae and 25.9% of multiparae.

**Table 2:** Striae gravidarum assessed by Davey’s scoring

	**n**	**Davey’s scores**			***p*-value**
		**0 (absent)**	**1-2 (mild)**	**3-8 (severe)**	
Primiparae (%)	94	68 (72.3)	22 (23.4)	4 (4.3)	0.000
Multiparae (%)	85	41 (48.2)	22 (25.9)	22 (25.9)	
Total (%)	179	109 (60.8)	44 (24.4)	26 (14.8)	

Data were analyzed using chi-squared test.

The QOL of pregnant women with and without striae gravidarum was evaluated (Table 3). There was no difference in WHOQOL-BREF scores between the presence and absence of striae gravidarum in primiparae and multiparae. Although primiparae did not show significant differences in scores on the three subcategories of Skindex-29 between the presence and absence of striae gravidarum, multiparae showed a significant difference in scores of emotion on Skindex-29 between them. In the whole group, scores of emotion on Skindex-29 among pregnant women with striae gravidarum were significantly higher than those without striae gravidarum.

**Table 3:** Skindex-29 and WHOQOL-BREF scores of pregnant women with and without striae gravidum

		**Striae gravidarum**			
		**Total**	**Absence**	**Presence**	***p*-value**
Total	n	179	109	70	
Skindex 29					
Emotion		2.5	2.5	5.0	0.012
		(0.0-10.0)	(0.0-7.5)	(0.0-18.1)	
Symptoms		14.3	14.3	17.9	0.163
		(10.7-25.0)	(10.7-21.4)	(10.7-28.6)	
Functioning		0.0	0.0	0.0	0.413
		(0.0-0.0)	(0.0-0.0)	(0.0-0.0)	
WHOQOL-BREF		3.5	3.5	3.6	0.342
		(3.2-3.8)	(3.2-3.9)	(3.2-3.8)	
Primiparae	n	94	68	26	
Skindex 29					
Emotion		2.5	2.5	6.3	0.173
		(0.0-10.0)	(0.0-7.5)	(0.0-20.6)	
Symptoms		17.9	14.3	19.6	0.307
		(10.7-25.0)	(10.7-24.0)	(10.7-28.6)	
Functioning		0.0	0.0	0.0	0.592
		(0.0-0.0)	(0.0-0.0)	(0.0-0.0)	
WHOQOL-BREF		3.5	3.6	3.5	0.703
		(3.3-3.8)	(3.3-3.8)	(3.3-3.9)	
Multiparae	n	85	41	44	
Skindex 29					
Emotion		2.5	0.0	5.0	0.011
		(0.0-8.8)	(0.0-5.0)	(0.0-16.9)	
Symptoms		14.3	14.3	16.1	0.193
		(10.7-25.0)	(10.7-19.6)	(10.7-25.0)	
Functioning		0.0	0.0	0.0	0.758
		(0.0-0.0)	(0.0-0.0)	(0.0-0.0)	
WHOQOL-BREF		3.5	3.5	3.6	0.526
		(3.2-3.8)	(3.2-4.0)	(3.2-3.8)	

Data were analyzed by Mann–Whitney *U* test.

( − ) indicates in interquartile range (IQR).

The QOL in each subgroup of severity in striae gravidarum was examined (Table 4). There were no differences in WHOQOL-BREF scores among the three subgroups. However, pregnant women with severe striae gravidarum showed significantly higher scores in emotion and functioning on Skindex-29 compared with those without striae gravidarum and those with mild striae gravidarum.

**Table 4:** Skindex-29 scores of pregnant women with striae gravidum by Davey’s scoring

	**Total**	**Davey’s scores**			***p*-value**
		**0 (absent)**	**1-2 (mild)**	**3-8 (severe)**	
n	179	109	44	26	0.000
Emotion	2.5	2.5^1)^	2.5^2)^	8.8^1)^^2)^	0.000
Total (%)	179	109 (60.8)	44 (24.4)	26 (14.8)	
	(0.0-10.0)	(0.0-7.5)	(0.0-14.4)	(2.5-25.0)	
Symptoms	14.3	14.3	14.3	21.4	0.163
	(10.7-25.0)	(10.7-21.4)	(10.7-25.0)	(13.4-28.6)	
Functioning	0.0	0.0^3)^	0.0^4)^	0.0^3)^^4)^	0.013
	(0.0-0.0)	(0.0-0.0)	(0.0-0.0)	(0.0-4.2)	
WHOQOL-BREF	3.5	3.5	3.6	3.4	3.4
	(3.2-3.8)	(3.2-3.9)	(3.3-3.8)	(3.2-3.8)	

^1)^*P* = 0.000 ^2)^*P =* 0.005 ^3)^*P* = 0.012 ^4)^*P* = 0.009.

Date were analyzed by Kruskal-Wallis test followed by Mann–Whitney test with Bonferroni correction.

( − ) indicates in interquartile range (IQR).

## Discussion

An assessment system for striae gravidarum has not been established. Generally applicable methods to estimate the striae gravidarum were reported by Davey [4] and Atwal et al. [16], and original rating systems are also found in some other studies. In Davey’s method, the striae gravidarum spreading through the whole abdomen are evaluated, and this method is widely used. An evaluation of color of the striae gravidarum as well as their presence features in Atwal’s method which was reported recently. As three researchers were to evaluate striae gravidarum independently in this study, the assessment system needed to be simple and easy to perform in order to reduce variations in measurement among researchers. We thus adopted Davey’s method in the present study. As a result, the prevalences of striae gravidarum in the three clinics were 39.4%, 36.7% and 42.0%, respectively, suggesting that Davey’s method is a stable rating system when multiple researchers perform it.

The prevalence of striae gravidarum among Japanese women was 39.1% (27.7% for primiparae and 51.8% for multiparae) in this study. This is a lower rate than in previous reports. The occurrence rates of striae gravidarum among primiparae in the UK were 48.6% in Davey's first report in 1970 [4] and 63% in 2001 [17]. Although some studies used Davey 's method, it is difficult to compare the prevalence of striae gravidarum because of a lack of data about parity [18,19] or unclear descriptions of the assessment procedures used [20].

Striae gravidarum are described in textbooks, without any evidence, as a serious concern during pregnancy [21]. However, there are no studies of the influence of striae gravidarum on the QOL of pregnant women because the striae gravidarum have conventionally been considered a minor medical concern. Once striae gravidarum appear during pregnancy, they remain after delivery. As we considered striae gravidarum to be an abnormal skin condition, the influence of striae gravidarum on women’s dermatology-specific QOL was also evaluated in the study.

In counseling for skin disease, the QOL evaluation is beneficial as information about the interests and concerns of subjects can be obtained [22]. It is desirable for a QOL evaluation of patients with skin disease to estimate the generic and dermatology-specific QOL at the same time. The combination of a generic QOL scale such as SF-36 or WHOQOL-100 and a dermatology-specific QOL scale such as Skindex-29 or Skindex-17 is most often used in such clinical studies [22]. Each scale has unique characteristics. An item about sexuality is not included in SF-36, whereas it is included in WHOQOL-100. We thought that the item about sexuality could be important in assessing the QOL of healthy pregnant women. Furthermore, we selected WHOQOL-BREF, i.e., a brief version of WHOQOL-100, in consideration of the time required for responding.

As for the score of WHOQOL-BREF, there were no significant differences according to the presence (median 3.6) or absence (median 3.5) of striae gravidarum in this study. The mean scores of WHOQOL-BREF were 3.5 ± 0.5 in pregnant women with striae gravidarum and 3.6 ± 0.4 in those without them. In the manual of the WHOQOL-BREF Japanese version, the general scores of WHOQOL-BREF are 3.3 ± 0.5 for 20–29 year-old and 3.2 ± 0.4 for 30–39 year-old Japanese women, suggesting that the participants’ generic QOL was not affected by the presence of striae gravidarum. However, scores of emotion in the whole group of pregnant women and multiparae were significantly increased by the occurrence of striae gravidarum, whereas those in primiparae were not (Table 3). As described in Tables 2 and 4, severe striae gravidarum appeared more frequently in multiparae than in primiparae and impaired emotion in the dermatology-specific QOL significantly more than mild striae gravidarum did. The median score of emotion in pregnant women with striae gravidarum was 5.0 and the mean score was 10.7. Mean scores of emotion reported elsewhere were 12.3 in patients with ectodermal dysplasia [23], 29.5 in vitiligo patients [24], and 48 in patients with cutaneous lupus erythematosus [25]. In free answers from the participants, pregnant women stated their reconciliation with this unpleasant state e.g., "I cannot help striae gravidarum because they are naturally caused by the pregnancy." or "Although I want to prevent the striae gravidarum if possible, I have given up on preventing the striae gravidarum because I can obtain greater things from pregnancy than striae gravidarum”. These results indicate that the striae gravidarum moderately affected emotion in the dermatology-specific QOL, similar to other skin conditions. It is therefore certain that striae gravidarum affect the emotions of pregnant women. In severe cases of acne [26] and vitiligo [24], significantly elevated scores of emotion on Skindex-29 were reported, which was similar to our results, suggesting that severity is also important when assessing the impact on emotion.

In scores of symptoms, there were no significant differences between the presence and absence of striae gravidarum, and the mean score was 18.6 in the whole group of pregnant women. The mean scores of symptoms were reported to be 16.3 and 25.3 in groups containing males and females with vitiligo [24] and ectodermal dysplasia [23]. Regardless of the presence or absence of striae gravidarum, pregnant women possibly suffer from such dermatological symptoms at similar levels to other skin diseases. As a result, most pregnant women might seek prophylaxis for striae gravidarum. In the study, 77.7% of pregnant women used ointment or lotion, expecting a prophylactic effect against striae gravidarum.

Scores of functioning in severe cases were significantly higher than mild cases and cases without striae gravidarum. However, the mean score (1.4) in the whole group of pregnant women was very low as compared with other skin diseases and is equivalent to the mean score of 4 reported in controls [26], suggesting that striae gravidarum are unlikely to affect the functioning of pregnant women. Striae gravidarum mainly appear on the abdomen, which can be covered with clothes and is rarely exposed to the public eye, unlike some other skin diseases, so it might not affect functioning. Skindex-17 consists of two subcategories; emotion and symptoms of Skindex-29, and might be appropriate to examine the influence of striae gravidarum on QOL.

### Limitations

This is the first trial to evaluate the QOL of pregnant women with striae gravidarum, and so no comparison of results among studies or calculation of an adequate sample size could be conducted. The sample size was based on those in the previous studies which used Skindex-29 [23-26].

The psychological influence of striae gravidarum on pregnant women seems to vary with race. Such psychological burdens are associated with QOL or several preventive behaviors. In this study, 77.7% (138/179) of Japanese pregnant women used skin lotions or skin creams in order to prevent striae gravidarum. As the relationship between QOL and preventive behavior by pregnant women with striae gravidarum in several countries is not obvious, such examinations might be needed in future studies.

## Conclusions

A point to be emphasized is the fact that striae gravidarum have a moderate affect on QOL in Japanese pregnant women similar to those of other chronic skin diseases, although striae gravidarum has been considered as a minor complaint. The occurrence of striae gravidarum influences the QOL of pregnant women, and their severity is important. In this study, 22 of 26 severe cases were multiparae and the emotion aspects of dermatology-specific QOL in multiparae were impaired by striae gravidarum. Judging from the perspective of the QOL of pregnant women, it is considered important to prevent severe striae gravidarum of multiparae, because the damage to QOL by the striae gravidarum could be relieved by keeping their severity to a mild level.

## Abbreviations

WHO: World Health Organization; QOL: Quality of life.

## Competing interests

The authors have no conflict of interest to declare. Osaka University Graduate School of Medicine provided funding and resources for this research. No additional funding was required for this research.

## Authors' contributions

KY considered and designed the concept of the study, collected, analyzed, and interpreted the data and drafted the manuscript. NS/KO participated in designing the concept of the study and drafted the manuscript. All authors approved the final manuscript.
